# Premature Brain Aging in Baboons Resulting from Moderate Fetal Undernutrition

**DOI:** 10.3389/fnagi.2017.00092

**Published:** 2017-04-11

**Authors:** Katja Franke, Geoffrey D. Clarke, Robert Dahnke, Christian Gaser, Anderson H. Kuo, Cun Li, Matthias Schwab, Peter W. Nathanielsz

**Affiliations:** ^1^Structural Brain Mapping Group, Department of Neurology, University Hospital JenaJena, Germany; ^2^Radiology, University of Texas Health Science Center San AntonioSan Antonio, TX, USA; ^3^Department of Psychiatry, University Hospital JenaJena, Germany; ^4^Texas Pregnancy and Life Course Health Research Center, Southwest National Primate Research Center, Texas Biomedical Research InstituteSan Antonio, TX, USA; ^5^Animal Science, University of WyomingLaramie, WY, USA; ^6^Department of Neurology, University Hospital JenaJena, Germany

**Keywords:** *BrainAGE*, developmental programming, *in vivo*, maternal nutrient restriction (MNR), machine learning, non-human primates, magnetic resonance imaging (MRI)

## Abstract

Contrary to the known benefits from a moderate dietary reduction during adulthood on life span and health, maternal nutrient reduction during pregnancy is supposed to affect the developing brain, probably resulting in impaired brain structure and function throughout life. Decreased fetal nutrition delivery is widespread in both developing and developed countries, caused by poverty and natural disasters, but also due to maternal dieting, teenage pregnancy, pregnancy in women over 35 years of age, placental insufficiency, or multiples. Compromised development of fetal cerebral structures was already shown in our baboon model of moderate maternal nutrient reduction. The present study was designed to follow-up and evaluate the effects of moderate maternal nutrient reduction on individual brain aging in the baboon during young adulthood (4–7 years; human equivalent 14–24 years), applying a novel, non-invasive neuroimaging aging biomarker. The study reveals premature brain aging of +2.7 years (*p* < 0.01) in the female baboon exposed to fetal undernutrition. The effects of moderate maternal nutrient reduction on individual brain aging occurred in the absence of fetal growth restriction or marked maternal weight reduction at birth, which stresses the significance of early nutritional conditions in life-long developmental programming. This non-invasive MRI biomarker allows further longitudinal *in vivo* tracking of individual brain aging trajectories to assess the life-long effects of developmental and environmental influences in programming paradigms, aiding preventive and curative treatments on cerebral atrophy in experimental animal models and humans.

## Introduction

Although, moderate dietary restriction during adulthood appears to the lengthen lifespan (Fontana et al., 2010), dietary restriction during prenatal life has been clearly demonstrated to have the opposite effect, i.e., being related to an altered, suboptimal development of structure and function of multiple organ systems, a shortened lifespan, and increased prevalence for chronic diseases in later life (Entringer et al., 2012; Schuurmans and Kurrasch, 2013; Tarry-Adkins and Ozanne, 2014; Rando and Simmons, 2015; Zambrano et al., 2015), as well as permanent impairments in brain structure and function (Morgane et al., 1993; Morley and Lucas, 1997; Olness, 2003; Grantham-McGregor and Baker-Henningham, 2005; Wainwright and Colombo, 2006; Walker et al., 2007; Benton and ILSI Europe, 2008; Antonow-Schlorke et al., 2011; Rodriguez et al., 2012; Keenan et al., 2013; Muller et al., 2014). Still, fetal undernutrition due to decreased nutrient delivery and micronutrient deficiency is a worldwide societal challenge, with a multiplicity of causes, like poverty, natural disasters, war, and cultural habits, but also maternal dieting, teenage pregnancies, pregnancies in women over 35 years of age, women suffering from hyperemesis gravidarum or placental insufficiency, as well as in multiple pregnancies (Black et al., 2008; Baker et al., 2009; Beard et al., 2009; Roseboom et al., 2011; Raznahan et al., 2012; Zhang et al., 2015).

However, human retrospective studies are subject to lifestyle and environmental confounds and do not readily allow isolation and control of individual variables presumed to cause specific long-term outcomes, such as variable degrees of prenatal or postnatal nutrition, maternal stress etc. (Symonds et al., 2000; Piras et al., 2014). Animal models that introduce controlled perturbations are required to determine, quantify, and understand the causal relationships between perinatal nutrient delivery and life-long effects on brain maturation and aging. Developmental programming studies have been mainly conducted in polytocous, altricial rodents, i.e., species with substantially different trajectories of fetal and neonatal brain development from monotocous, precocial mammals, including humans (Ganu et al., 2012; Fontana and Partridge, 2015). Non-human primates have many similarities in physiology, neuroanatomy, reproduction, development, cognition, and social complexity to humans (Vandeberg et al., 2009; Phillips et al., 2014). The baboon is an old world primate, which is the closest available species to relate to human programming in terms of reproduction, developmental physiology, gene function, or brain structure (Vandeberg et al., 2009; Atkinson et al., 2015).

To enable studies directed at translation to determine the effects of malnutrition in humans we have developed a baboon model of 30% reduction in global maternal nutrition during pregnancy, while controlling for all other psychosocial stressors. In the maternal nutrient restriction (MNR) fetus, we have already shown an altered trajectory of brain development (Antonow-Schlorke et al., 2011). Subsequently, the adolescent MNR baboon offspring showed altered postnatal cognitive and behavioral performances at 3.3 years of age (human equivalent 11.5 years) (Rodriguez et al., 2012; Keenan et al., 2013). To further track the life-long consequences of MNR in neuroanatomical maturation and aging we aimed to develop an *in vivo*, non-invasive biomarker for brain aging in order to capture individual deviations in the MNR offspring, which will translate and be comparable to humans. Therefore, this study utilizes a baboon-specific adaption of our innovative *BrainAGE* method, recently developed for modeling brain aging in human samples. The *BrainAGE* method is based on a fully-automatic preprocessing pipeline for structural *in vivo* brain magnetic resonance imaging (MRI) data and uses pattern recognition methods to evaluate individual brain aging (Franke et al., 2010, 2012). In humans, it has already been validated and applied in several studies, indicating subtle deviations in age-related brain structure due to various health and lifestyle conditions, with premature brain aging being related to cognitive decline and clinical symptoms (Franke et al., 2012, 2014; Gaser et al., 2013).

For the present study, a novel, fully automatic preprocessing pipeline for baboon brain MRI data was developed and sex-specific reference curves of baboon brain tissue volumes across the adult lifespan were constructed, based on non-invasive *in vivo* MRI data from 29 control subjects (aged 4–22 years; human equivalent 14–77 years). Second, we present a species-specific adaptation of our *BrainAGE* method based on pattern recognition methods to evaluate individual brain age in the baboon. Finally, we employed this non-invasive, non-terminal *in vivo* biomarker to study structural brain aging in MNR and control offspring at about 5 years of age (human equivalent 17.5 years). We hypothesized that moderate MNR during pregnancy would lead to premature neuroanatomical aging in the young adult offspring. In line with the sexual dimorphism hypothesis in Developmental Programming, we hypothesized that effects of decreased fetal nutrient delivery on individual brain aging would differ between male and female offspring (Aiken and Ozanne, 2013).

## Materials and methods

### Subjects and MRI scanning

The study included two samples. The lifespan-sample, which was used to analyze changes in brain tissue across adulthood as well as to build and test the baboon-specific brain age estimation model, included 29 (15 female) healthy control subjects (*Papio hamadryas*), aged 4–22 years (mean age 9.5 ± 4.9 years) (Table 1), which is equivalent to 14–77 years in humans. A second sample of 11 subjects (5 female) formed the experimental group of subjects with fetal undernutrition due to MNR (see Production of MNR and CTR Offspring). 12 same-aged subjects (5 female) from the lifespan-sample were included in the control (CTR) group. At time of MRI data acquisition, MNR and CTR subjects were aged 4–7 years (Table 2), which is equivalent to 14–24 years in humans. Each subject was scanned on a 3 Tesla whole body MRI scanner (TIM Trio, Siemens Medical Solutions, Malvern, PA), using a T1-weighted sequence.

**Table 1:** **Morphologic data in the lifespan-sample**.

	**Females**	**Males**	***F* statistic**
*n*	15	14	–
Age at MR scan (years)	9.43 ± 3.89	9.62 ± 6.02	*F* = 0.0 (*p* = 0.92)
Absolute GM volume (ml)	87.3 ± 8.6	95.9 ± 8.8	***F*** = **7.0 (*****p*** = **0.01)**
Absolute WM volume (ml)	60.5 ± 7.0	67.4 ± 5.3	***F*** = **8.9 (*****p*** = **0.006)**
Absolute CSF volume (ml)	38.4 ± 4.9	46.5 ± 5.1	***F* = 19.1 (*p* < 0.001)**
Absolute TIV (ml)	186.7 ± 14.4	210.4 ± 14.0	***F*** = **20.1 (*****p*** < **0.001)**
Fractional GM volume (/TIV)	0.47 ± 0.03	0.46 ± 0.02	*F* = 1.4 (*p* = 0.25)
Fractional WM volume (/TIV)	0.32 ± 0.02	0.32 ± 0.02	*F* = 0.1 (*p* = 0.73)
Fractional CSF volume (/TIV)	0.21 ± 0.02	0.22 ± 0.02	*F* = 3.4 (*p* = 0.08)

*Data are displayed as mean ± standard deviation (SD). Bold type indicates statistical significance*.

**Table 2:** **Descriptive statistics for morphologic data and test results in subjects with maternal nutrient restriction (MNR) and ***ad lib*** fed controls (CTR)**.

	**Female**			**Male**			**Total**		
	**CTR**	**MNR**	***F* statistic**	**CTR**	**MNR**	***F* statistic**	**CTR**	**MNR**	***F* statistic**
*n*	5	5	–	7	6	–	12	11	–
Birth weight (kg)	0.87 ± 0.12	0.78 ± 0.15	*F* = 1.2 (*p* = 0.30)	0.90 ± 0.10	0.83 ± 0.09	*F* = 1.7 (*p* = 0.22)	0.89 ± 0.10	0.81 ± 0.11	*F* = 3.2 (*p* = 0.09)
Weight at MR scan (kg)	12.72 ± 1.53	13.90 ± 0.98	***F*** = **7.2 (*****p*** = **0.03)**	17.91 ± 5.56	20.77 ± 4.65	*F* = 0.5 (*p* = 0.48)	15.75 ± 4.99	17.65 ± 4.90	*F* = 1.7 (*p* = 0.21)
Age at MR scan (years)	4.75 ± 0.76	4.38 ± 0.20	*F* = 1.1 (*p* = 0.21)	5.00 ± 1.34	5.55 ± 1.38	*F* = 0.5 (*p* = 0.21)	4.90 ± 1.09	4.97 ± 1.11	*F* = 0.0 (*p* = 0.21)
Absolute GM volume (ml)	90.1 ± 6.9	86.4 ± 4.4	*F* = 0.9 (*p* = 0.36)	96.0 ± 10.1	99.0 ± 13.2	*F* = 0.1 (*p* = 0.74)	96.6 ± 8.6	93.3 ± 11.8	*F* = 0.7 (*p* = 0.43)
Absolute WM volume (ml)	55.6 ± 8.1	53.2 ± 4.6	*F* = 0.5 (*p* = 0.48)	65.1 ± 5.6	65.1 ± 8.6	*F* = 0.0 (*p* = 0.89)	59.7 ± 9.2	61.2 ± 8.0	*F* = 0.2 (*p* = 0.62)
Absolute CSF volume (ml)	35.0 ± 2.0	41.8 ± 4.4	***F*** = **6.9 (*****p*** = **0.03)**	46.5 ± 5.9	41.8 ± 3.0	*F* = 2.6 (*p* = 0.14)	41.7 ± 7.5	41.8 ± 3.5	*F* = 0.0 (*p* = 0.97)
TIV (ml)	181.3 ± 14.6	195.4 ± 21.5	*F* = 0.9 (*p* = 0.90)	213.7 ± 14.3	206.7 ± 22.4	*F* = 0.4 (*p* = 0.53)	200.2 ± 21.6	195.4 ± 21.5	*F* = 0.3 (*p* = 0.57)
Fractional GM volume (/TIV)	0.50 ± 0.01	0.48 ± 0.01	***F*** = **7.9 (*****p*** = **0.03)**	0.47 ± 0.01	0.48 ± 0.02	*F* = 0.4 (*p* = 0.53)	0.48 ± 0.01	0.48 ± 0.02	*F* = 1.1 (*p* = 0.31)
Fractional WM volume (/TIV)	0.31 ± 0.02	0.29 ± 0.01	*F* = 1.7 (*p* = 0.23)	0.31 ± 0.02	0.32 ± 0.01	*F* = 1.1 (*p* = 0.32)	0.31 ± 0.02	0.30 ± 0.02	*F* = 0.1 (*p* = 0.81)
Fractional CSF volume (/TIV)	0.19 ± 0.02	0.23 ± 0.02	*F* = 5.3 (*p* = 0.05)	0.22 ± 0.02	0.20 ± 0.02	*F* = 1.0 (*p* = 0.33)	0.21 ± 0.02	0.22 ± 0.02	*F* = 0.7 (*p* = 0.42)
*BainAGE* score (years)	−1.58 ± 1.36	1.16 ± 0.80	***F*** = **11.4 (*****p*** = **0.01)**	0.86 ± 1.54	0.89 ± 2.43	*F* = 0.0 (*p* = 0.99)	−0.16 ± 1.89	1.01 ± 1.80	*F* = 2.2 (*p* = 0.15)

*Data are displayed as mean ± standard deviation (SD). Bold type indicates statistical significance*.

### Production of MNR and CTR offspring

Female baboons were housed in harem groups of 16 females and one vasectomized male at the Southwest National Primate Research Center at San Antonio, Texas, USA. Groups of mothers that eventually gave birth to the MNR and CTR offspring were socialized in the presence of a vasectomized male while eating Purina Monkey Diet 5038 (Purina, St. Louis, Missouri, USA) containing crude protein not less than 15%, crude fat not less than 5%, crude fiber not more than 6%, ash not more than 5% and added minerals not more than 3% *ad libitum*. The management of the feeding of *ad libitum* and nutrient reduction has previously been described in detail (Keenan et al., 2013). Following acclimation, the vasectomized male was replaced by a proven breeder male. Timing of pregnancy was performed by following sex-skin turgescence (Hendrickx and Peterson, 1997). Following confirmation of pregnancy by ultrasound at 30 days gestation, baboons with moderate MNR received 70% of the average daily amount of feed eaten by the *ad libitum* control females on a weight adjusted basis at the same gestational age. Water was continuously available. Mothers were of similar age (mean age ± *SD:* 11.5 ± 0.51 years) and morphometric phenotype. One cage was randomly selected for *ad libitum* feeding on normal primate feed pellets and one cage for mothers fed 70% of the feed eaten by control females on a weight-adjusted basis from the time of diagnosis of pregnancy (~30 days gestation) for the rest of pregnancy and through lactation. Initially, 40 adult females were recruited to the study, with 18 mothers-to-be being placed on the reduced diet and 22 on *ad libitum* feed. All mothers delivered spontaneously at full term. In the whole cohort, male CTR offspring of *ad lib* fed mothers weighed 930 ± 40 g (mean ± SEM; *n* = 10) at birth, male MNR offspring weighed 820 ± 39 g (*n* = 9; *p* < 0.05). Female CTR neonates weighed 820 ± 40 g (*n* = 12), female MNR neonates weighed 730 ± 40 g (*n* = 9; *p* < 0.05). Weights of the randomly chosen MRI subsample used for the present study were within the same range as the whole sample (Table 1). All offspring were reared with their mothers in group-based housing until 9 months of age. Juvenile offspring were transferred to the University of Texas Health Science Center at San Antonio in cohorts of 5–7 subjects over a 9-months period and housed individually in the visual and auditory presence of ≥ 6 other peers in the Laboratory Animal Resources facility.

All animal procedures were performed in accordance with accepted standards of humane animal care approved by the Texas Biomedical Research Institute and University of Texas Health Science Center at San Antonio Institutional Animal Care and Use Committees and conducted in facilities approved by Association for Assessment and Accreditation of Laboratory Animal Care International Inc (AAALAC).

### Basic concept of the brain age estimation framework

We recently developed the *brain age gap estimation* (*BrainAGE*) framework to model healthy human brain aging (Franke et al., 2010). Its basic concept is the aggregation of the complex, multidimensional aging pattern across the whole brain into one single value, i.e., the estimated brain age. In human samples, the *BrainAGE* framework accurately and reliably estimates the age of individual brains with minimal preprocessing and parameter optimization using anatomical MRI scans (Franke et al., 2010, 2012). It also has the potential to identify pathological brain aging on an individual level (Franke et al., 2012; Gaser et al., 2013).

**Figure 1:**
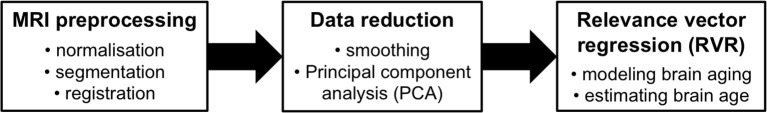
**General flowchart of the brain age estimation framework**.

In general, the process includes three steps (Figure 1). First, the raw T1-weighted image data are preprocessed with a standardized voxel-based morphometry (VBM) pipeline. Second, data reduction is performed on the preprocessed MRI data in order to reduce computational costs, to avoid severe over-fitting, as well as to produce a robust and widely applicable age estimation model. Third, relevance vector regression (RVR) is utilized to capture the multidimensional aging patterns across the whole brain in order to model brain aging over a wide age range and to subsequently estimate individual brain ages. In the present study, the brain age estimation framework includes a novel baboon-specific MRI preprocessing pipeline (see Preprocessing of MRI Data) as well as building the new species-specific model of healthy brain aging in baboons (see The *BrainAGE* Method and Its Baboon-specific Adaptation).

### Preprocessing of MRI data

**Figure 2:**
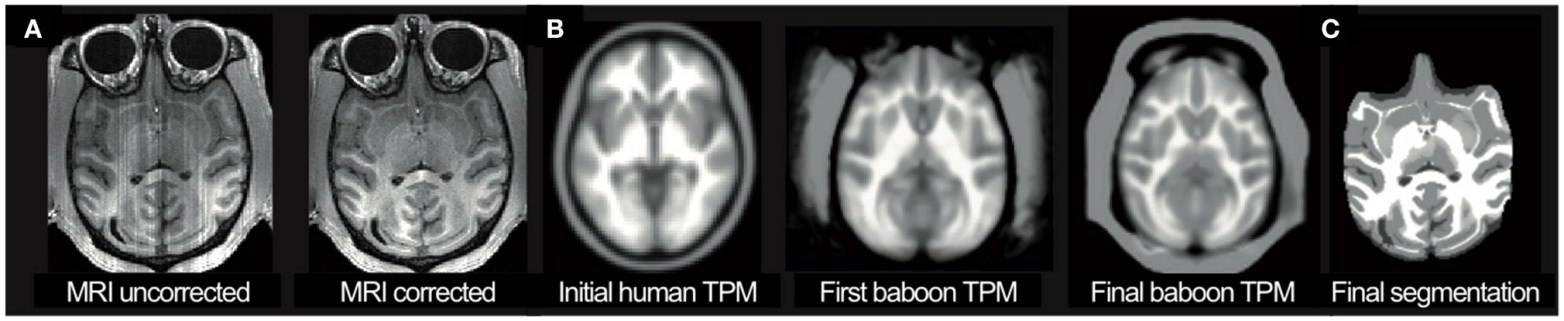
**(A)** Shown are the original T1-weighted image and the slice-corrected version. **(B)** For the segmentation process, a baboon specific tissue probability map (TPM; shown as label map) was used in an iterative process, starting with a scaled human template (left) and refinements during each iteration. **(C)** The final baboon TPM was used to create the final segmentation of the individual MRI.

T1-weighted image MR image data we acquired using a 3D acquisition scheme with in-plane images in the sagittal orientation in order to maximize isotropic resolution while avoiding aliasing artifacts. This acquisition strategy necessitated the use of slice-based inhomogeneity correction to remove MR protocol dependent slice artifacts (Figure 2A) (Van Leemput et al., 1999; Cohen et al., 2000). Then, a spatial adaptive non-local means (SANLM) filter (Christidis and Cox, 2006) was applied to reduce high-frequency noise. For segmentation and spatial registration a baboon-specific tissue probability map (TPM) and a “Diffeomorphic Anatomical Registration using Exponentiated Lie algebra” (DARTEL) template (Ashburner, 2007) were required. The template was created in an iterative process based on a rescaled human template (Figure 2B). For initialization, an affine transformation was used to scale the human SPM12 TPM and the VBM12 Dartel template map to the expected brain size of baboons. Image resolution of the template was changed to isotropic voxel size of 0.75 mm. For each iteration step, the resulting tissue maps were averaged and smoothed with a full-width-at-half-maximum (FWHM) kernel of 2 mm to estimate an affine registration in order to create a new TPM, T1 average map and brain mask. For averaging data, the median function was used to reduce distortions by outliers and failed processing. Iterations were stopped if the change compared to the previous template was below a pre-defined threshold, resulting in the final segmentation (Figure 2C).

### Data reduction

Preprocessed MRI data were smoothed with a 3-mm FWHM smoothing kernel and images were resampled to 3 mm. Data were further reduced by applying principal component analysis (PCA).

### The *BrainAGE* method and its baboon-specific adaptation

The brain age estimation framework uses RVR, which was introduced as a Bayesian alternative to support vector machines (SVM) for obtaining sparse solutions to pattern recognition tasks (Tipping, 2000, 2001). Former results indicated favorable performance of RVR to capture the typical age-specific atrophy patterns across the whole brain (Franke et al., 2010). A linear kernel was chosen since age estimation accuracy is not improving when choosing non-linear kernels (Franke et al., 2010). Besides and in contrast to the use of support vector machines, parameter optimization during the training procedure is not necessary. More details can be found in (Franke et al., 2010).

**Figure 3:**
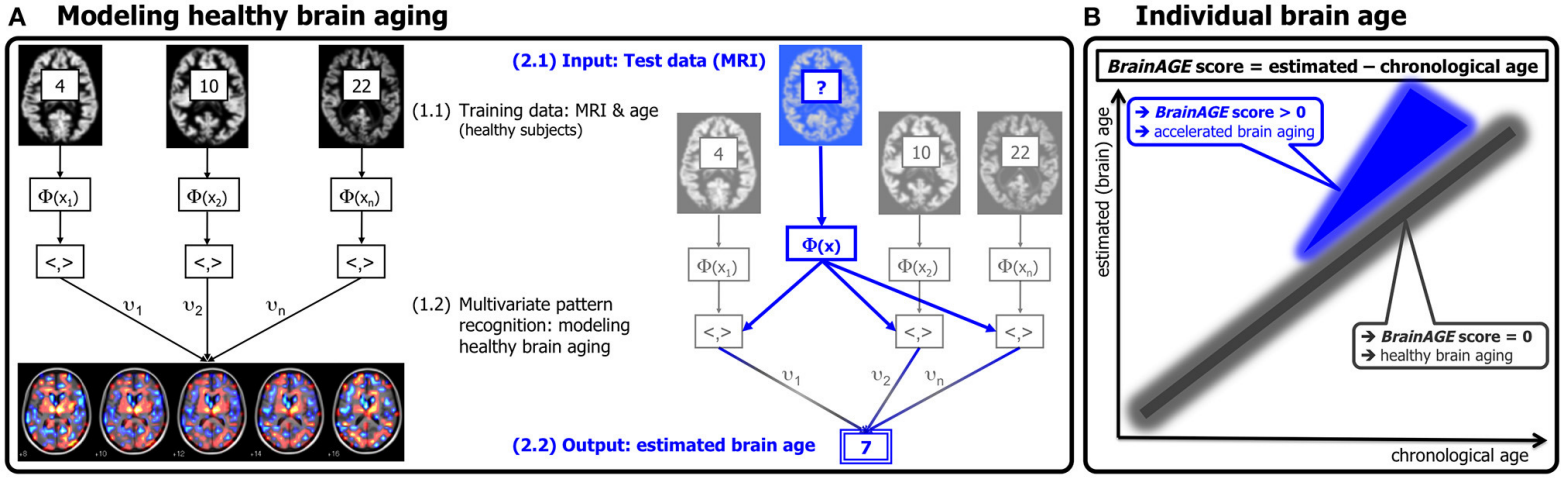
**Depiction of the original brain age estimation framework for humans. (A)** The model of healthy brain aging is trained with the chronological age and preprocessed structural MRI data of a training sample (left; with an exemplary illustration of the most important voxel locations that were used by the age regression model). Subsequently, the individual brain age of a previously unseen test subject is estimated, based on MRI data (blue; picture modified from Schölkopf and Smola, 2002). **(B)** The difference between the estimated and chronological age results in the deviation (i.e., *BrainAGE*) score. Consequently, positive deviation scores indicate accelerated brain aging. (Image reproduced from Franke et al., 2012, with permission from Hogrefe Publishing, Bern).

In general, the model is trained with preprocessed whole brain structural MRI data as well as the corresponding chronological ages of a training sample, resulting in a complex model of brain aging (Figure 3A, left panel). Put in other words, the algorithm uses those whole-brain MRI data from the training sample that represent the prototypical examples within the specified regression task (i.e., brain aging). Besides, voxel-specific weights can be calculated that represent the importance of each voxel within the specified regression task (i.e., brain aging).

Subsequently, the brain age of a test subject can be estimated using the individual tissue-classified MRI data, aggregating the complex, multidimensional aging pattern across the whole brain into one single value (Figure 3A, right panel). In other words, all the voxels of the test subject's MRI data are weighted by applying the voxel-specific weighting matrix. Then, the brain age is calculated by applying the regression pattern of healthy brain aging and aggregating all voxel-wise information across the whole brain. The difference between estimated brain age and the true chronological age will reveal an individual deviation score, namely the *brain age gap estimation* (*BrainAGE*) *score*. Consequently, positive or negative values of this deviation score directly quantify the amount of acceleration or deceleration in individual brain aging, respectively (Figure 3B).

Here, gray matter (GM) images resulting from the baboon-specific preprocessing pipeline described above were used to build the model of neuroanatomical aging in baboons. The brain age estimation model was trained and tested via leave-one-out cross-validation, i.e., preprocessed MRI data from 28 out of 29 baboons was used for training and the brain age of the left-out subject was estimated subsequently. This procedure was repeated 29 times. Brain ages in the MNR subjects were calculated using the whole lifespan sample as the training sample.

### Technical notes

The whole brain age estimation framework works fully automatically. All MRI preprocessing, data reduction, model training, and brain age estimation is done using Matlab. Preprocessing of the *in vivo* T1-weighted images was done using the toolboxes “Statistical Parametric Mapping” (SPM12; http://www.fil.ion.ucl.ac.uk/spm) and our new “Computational Anatomy Toolbox for SPM” (CAT12; http://dbm.neuro.uni-jena.de). PCA is performed using the “Matlab Toolbox for Dimensionality Reduction” (http://ict.ewi.tudelft.nl/~lvandermaaten/Home.html). To compute the age regression model as well as to predict the individual brain ages, the freely available toolbox “The Spider” (http://www.kyb.mpg.de/bs/people/spider/main.html) is used.

Baboon TPM and template generation takes around 30 min per subject and iteration, ending up in about 48 h for the whole sample. For modeling healthy brain aging with RVR, one cross-validation loop in the life-span sample of 29 subjects takes between 0.2 and 1.2 s on MAC OS X, Version 10.6.8, 2.8 GHz Intel Core 2 Duo. Thus, the whole process of training the baboon-specific brain age estimation model and subsequent estimation of individual brain age for all 29 subjects takes about 20 s in total.

### Statistical analyses

First, volumes of GM, white matter (WM), cerebrospinal fluid (CSF), and total intracranial volume (TIV) were analyzed using regression models. To test whether age effects were significantly associated with brain volumes, *F* statistics of linear and quadratic regression models were compared. To measure the accuracy of the brain age estimation model, the correlation coefficient between chronological and estimated brain age as well as the mean absolute error (MAE) was calculated:

(1) $$\begin{array}{r} \text{MAE}=1/{\text{n}}^{*}\Sigma_{\text{i}}|{\text{BA}}_{\text{i}}-{\text{CA}}_{\text{i}}| \end{array}$$

with *n* being the number of subjects in the test sample, *CA*_*i*_ the chronological age, and *BA*_*i*_ the structural brain age estimated by the model. Best-fit was tested comparing *F* statistics of linear and quadratic regression models.

Before analyzing brain aging in MNR baboons, birth weight, weight and age at time of MR scan, as well as brain volumes were compared between MNR and CTR groups using analysis of variance (ANOVA). As the range of chronological ages was relatively broad (4–7 years), age was included as a covariate (except for birth weight). Effect size was calculated using partial η^2^. To analyze differences in individual brain aging between both groups, the individual deviation scores, i.e., the *BrainAGE* scores, were calculated:

(2) $$\begin{array}{l} BrainAGE\text{ score}={\text{BA}}_{\text{i}}-{\text{CA}}_{\text{i}} \end{array}$$

We have previously reported sex differences in neurodevelopment and cognitive performance for the same cohort of MNR baboon (Rodriguez et al., 2012; Keenan et al., 2013). Therefore, we also tested the effect of sex on structural brain aging. Female and male offspring of mothers in the control group are referred as CTR females and CTR males, respectively, and female and male offspring of mothers in the moderate MNR group are referred as MNR females and MNR males, respectively. All statistical testing was performed using Matlab 7.11.

## Results

### Baboon brain volumes across adulthood

The lifespan-sample of healthy control subjects used to model healthy brain aging in baboon was aged 4–22 years at the time of data acquisition. Mean age did not differ between males and females (Table 1). Evaluation of *in vivo* MRI revealed that absolute GM volume, absolute WM volume, absolute CSF, as well as TIV were significantly higher in male compared to female subjects (Table 1). Absolute neocortical GM volume declined significantly with age, especially in males (Table 3). Absolute CSF volume increased with age only in females. Absolute WM volume as well as TIV did not vary with age.

**Table 3:** **Sex-specific regression models with linear and quadratic fit for age effects in absolute and fractional (/TIV) baboon brain volumes across adulthood**.

**Brain volume**	**Regression model**	**Female sample (*****n*** = **15)**		**Male sample (*****n*** = **14)**		**Whole lifespan-sample (*****n*** = **29)**	
		**Adjusted *R*^2^**	***F* statistic**	**Adjusted *R*^2^**	***F* statistic**	**Adjusted *R*^2^**	***F* statistic**
Absolute GM (ml)	Linear	0.19	*F* = 4.32 (*p* = *0.06*)	**0.47**	***F*** = **12.75 (*****p*** = **0.004)**	0.27^*^	***F*** = **11.15 (*****p*** = **0.002)**
	Quadratic	0.18	*F* = 2.49 (*p* = 0.12)	0.55^*^	***F*** = **8.85 (*****p*** = **0.005)**	**0.24**	***F*** = **5.37 (*****p*** = **0.01)**
Absolute WM (ml)	Linear	0.06	*F* = 1.89 (*p* = 0.19)	0.12	*F* = 2.72 (*p* = 0.12)	0.08	*F* = 3.31 (*p* = 0.06)
	Quadratic	0.20	*F* = 2.78 (*p* = 0.10)	0.20	*F* = 2.63 (*p* = 0.12)	0.05	*F* = 1.72 (*p* = 0.20)
Absolute CSF (ml)	Linear	**0.29**	***F*** = **6.75 (*****p*** = **0.02)**	−0.08	*F* = 0.01 (*p* = 0.93)	0.00	*F* = 0.88 (*p* = 0.36)
	Quadratic	0.33^*^	***F*** = **4.52 (*****p*** = **0.03)**	−0.02	*F* = 0.85 (*p* = 0.45)	0.09	*F* = 2.33 (*p* = 0.12)
TIV (ml)	Linear	−0.07	*F* = 0.07 (*p* = 0.80)	0.02	*F* = 1.21 (*p* = 0.29)	−0.03	*F* = 0.25 (*p* = 0.62)
	Quadratic	−0.06	*F* = 0.62 (*p* = 0.55)	−0.06	*F* = 0.64 (*p* = 0.55)	0.02	*F* = 1.24 (*p* = 0.31)
Fractional GM (/TIV)	Linear	0.85^*^	***F*** = **58.03 (*****p*** < **0.001)**	**0.92**	***F*** = **52.22 (*****p*** < **0.001)**	**0.65**	***F*** = **52.22 (*****p*** < **0.001)**
	Quadratic	**0.84**	***F*** = **26.33 (*****p*** < **0.001)**	0.94^*^	***F*** = **33.84 (*****p*** < **0.001)**	0.70^*^	***F*** = **33.84 (*****p*** < **0.001)**
Fractional WM (/TIV)	Linear	0.36^*^	***F*** = **6.56 (*****p*** = **0.03)**	**0.67**	***F*** = **19.53 (*****p*** = **0.002)**	**0.50**	***F*** = **28.91 (*****p*** < **0.001)**
	Quadratic	0.29	*F* = 3.06 (*p* = 0.10)	0.75^*^	***F*** = **14.24 (*****p*** = **0.003)**	0.51^*^	***F*** = **15.47 (*****p*** < **0.001)**
Fractional CSF (/TIV)	Linear	0.31^*^	***F*** = **5.55 (*****p*** = **0.04)**	−0.05	*F* = 0.56 (*p* = 0.47)	0.09	*F* = 3.73 (*p* = 0.06)
	Quadratic	0.29	*F* = 3.01 (*p* = 0.11)	0.08	*F* = 1.41 (*p* = 0.31)	0.09	*F* = 2.35 (*p* = 0.11)

*Asterisk indicates best-fit model. Bold type indicates significance*.

**Figure 4:**
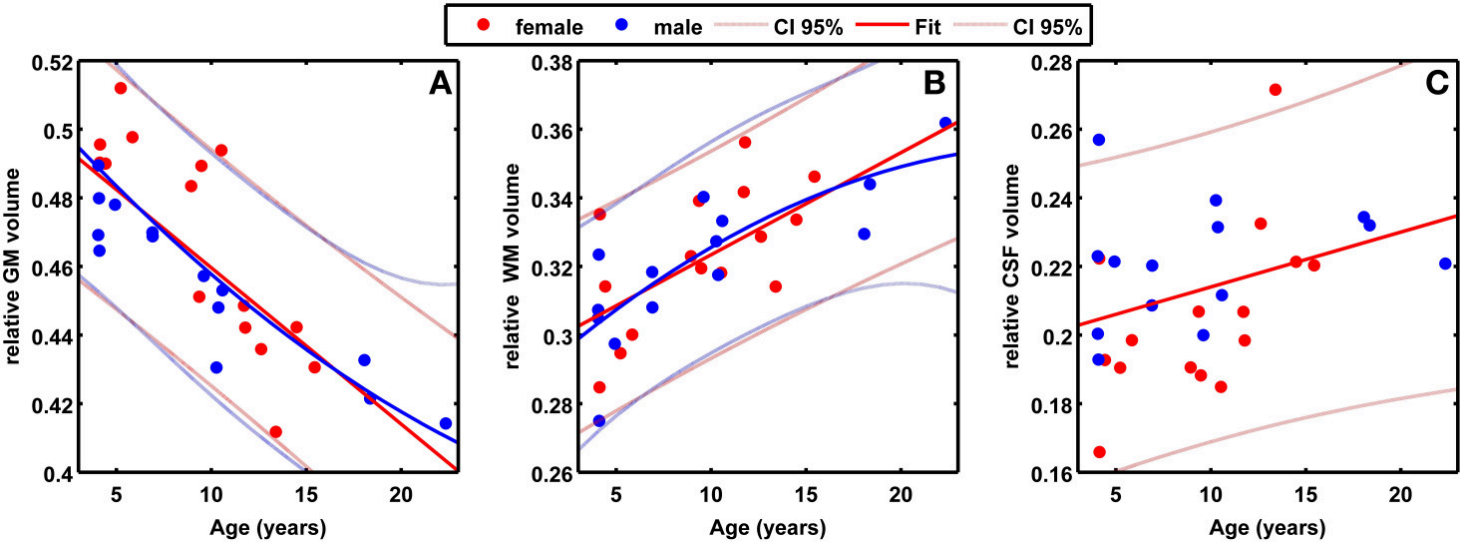
**(A)** Scatter plot of fractional GM volume (/TIV) against age (in years) for 29 healthy control baboons in the lifespan-sample (females in red, males in blue). The best fitting regression lines including the 95% confidence intervals (linear for females, quadratic for males) are superimposed. **(B)** Scatter plot of fractional WM volume (/TIV) against age, with linear regression curve for females and quadratic regression curve for males. **(C)** Scatter plot of fractional CSF volume (/TIV) against age, with linear regression curve for females.

To analyze characteristics of baboon brain tissue volumes during adulthood independent of individual differences in brain size, absolute brain volumes were corrected for TIV, resulting in individual proportions of GM, WM, and CSF in relation to individual TIV. Fractional GM, WM, and CSF volumes did not differ between genders (Table 1). In males and females, the GM decline was strongly explained by age (Figure 4A; Table 3), with a linear age effect in females (adjusted *R*^2^ = 0.85; *p* < 0.001) and a quadratic age effect in males (adjusted *R*^2^ = 0.94; *p* < 0.001). Fractional WM volume increased with age (Figure 4B; Table 3), with males showing a stronger relationship (quadratic fit: adjusted *R*^2^ = 0.75; *p* < 0.01) than females (linear fit: adjusted *R*^2^ = 0.36; *p* < 0.05). Fractional CSF volume showed a moderate increase with age only in females (linear fit: adjusted *R*^2^ = 0.31; *p* < 0.05), but not in males (Figure 4C; Table 3).

### Baboon brain age estimation model

**Figure 5:**
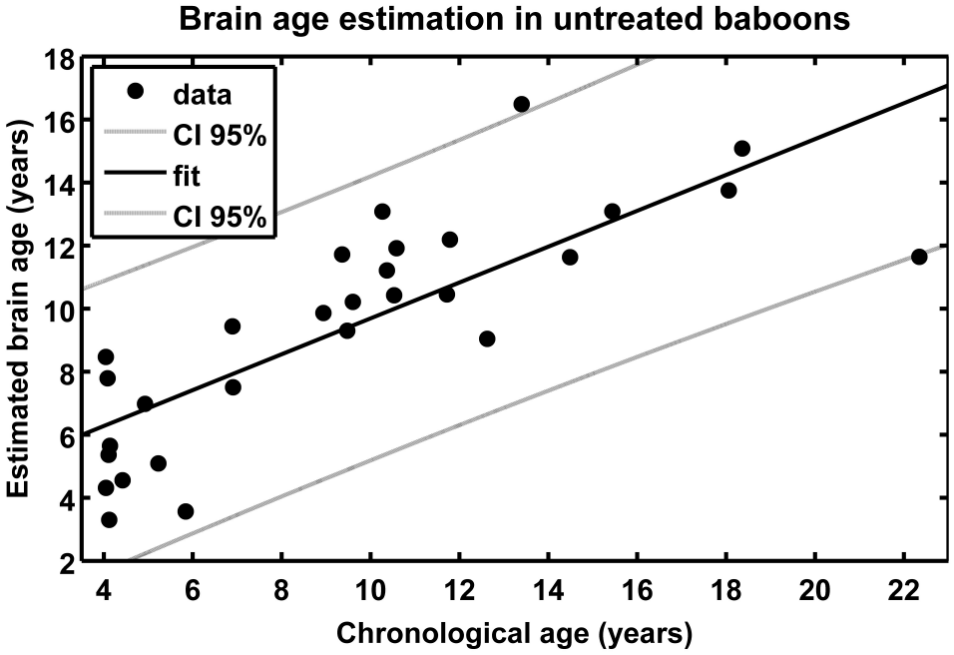
**Scatterplot of estimated brain age against chronological age (in years) resulting from leave-one-out cross-validation in 29 healthy control baboons using their ***in vivo*** anatomical MRI scans**. The overall correlation between chronological age and estimated brain age is *r* = 0.80 (*p* < 0.001), with an overall MAE of 2.1 years.

The baboon-specific brain age estimation model included a baboon-specific preprocessing pipeline for *in vivo* anatomical MRI scans and a machine-learning algorithm for pattern recognition in order to model a reference curve for normal aging of the baboon brain. Using preprocessed GM images, leave-one-out cross-validation in the whole lifespan-sample of healthy control subjects resulted in a correlation of *r* = 0.80 (*p* < 0.001; Figure 5) between chronological age and estimated brain age, with age estimation being slightly more accurate in females (*r* = 0.88) than in males (*r* = 0.81). The linear regression model resulted in the best fit (adjusted *R*^2^ = 0.62; *F* = 47.6; *p* < 0.001). The mean MAE between chronological age and estimated brain age was 2.1 years for the whole modeling sample of healthy control subjects (females: MAE = 1.5 years; males: MAE = 2.8 years).

### Morphometric characteristics of MNR model baboon offspring

In our whole non-human primate (NHP) model, MNR decreased birth weight (Li et al., 2013a). Birth weights of the MRI subsample in the present study, including a total of 11 randomly chosen offspring (5 female) with MNR and 12 randomly chosen control offspring (5 female) from mothers receiving full diet during pregnancy, fell within the same range as the total sample (Li et al., 2013a), with MNR offspring showing the tendency to weigh less than CTR offspring [*F*_(1, 18)_ = 3.2; *p* = 0.09; Table 2]. At time of *in vivo* MRI data acquisition, subjects were aged 4–7 years (human equivalent 14–24 years) (Table 2). Chronological age did not differ between experimental groups [*F*_(1, 18)_ = 0.1; *n.s*.] or gender [*F*_(1, 18)_ = 2.9; *n.s*.]. At the time of the MRI scan, female MNR offspring weighed more than female CTR offspring [*F*_(1, 7)_ = 7.2; *p* < 0.05; η^2^ = 0.51], showing an altered postnatal growth profile as a result of programming. No differences in weight at time of MRI scan were found in males (Table 2).

In the sample of MNR and CTR offspring, absolute as well as relative brain volumes did not differ between experimental groups. However, absolute CSF volume was increased in female MNR offspring as compared to female CTR offspring. Even more interesting, fractional GM volume corrected for individual TIV was significantly decreased in female MNR offspring [*F*_(1, 7)_ = 7.9; *p* < 0.05; η^2^ = 0.50; Table 2].

### Brain aging in adult MNR baboon

**Figure 6:**
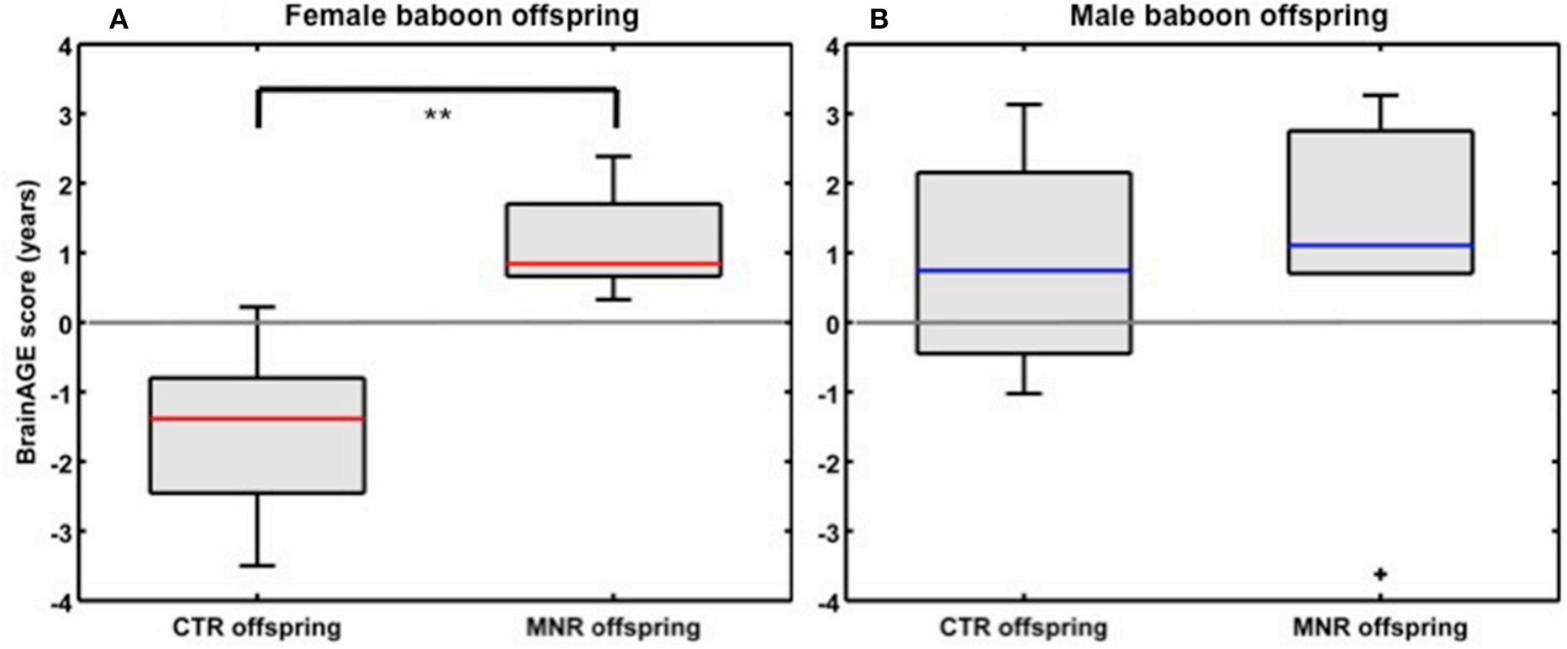
**Neurostructural aging in MNR model baboon offspring**. *BrainAGE* scores differed significantly between **(A)** young adult female CTR and MNR offspring by 2.74 years, **(B)** but not between male CTR and MNR offspring. The boxes contain values between the 25th and 75th percentiles of the groups, including the median (red/blue lines). Black lines extending above and below each box symbolize data within 1.5 times the interquartile range. Black “+” denotes outliers. Width of the boxes depends on group size. ^**^p = 0.01.

Baboon *BrainAGE* scores based on species-specific preprocessed GM images, which quantify baboon-specific neuroanatomical aging, were significantly increased by 2.74 years in young adult female MNR subjects as compared to young adult female CTR offspring [*F*_(*1, 7*)_ = 11.4; *p* = 0.01; η^2^ = 0.62; Figure 6A; Table 2], suggesting premature brain aging in female MNR offspring as a result of developmental programming due to fetal undernutrition. In males, *BrainAGE* scores did not differ between MNR and CTR offspring [*F*_(1, 9)_ = 0.0; *n.s*.; Figure 6B; Table 2].

## Discussion

To our knowledge this is the first study to combine the power of *in vivo* MRI data acquisition and the development of a controlled NHP model of developmental programming to follow the effects on structural brain development and aging. The translational power of these studies is shown by a parallel MRI study in the Dutch famine birth cohort, in which decreased total brain volume in late adulthood was already shown in those who had been undernourished prenatally (de Rooij et al., 2016). Further research in the Dutch famine birth cohort will investigate whether exposure to fetal undernutrition during early gestation has an effect on individual brain aging in late-life. This present study provides important novel insights into experimental brain programming using the closest available species with regards to human programming to model (experimentally induced) brain changes. First, we developed a novel fully-automatic baboon-specific MRI data preprocessing pipeline, based on and comparable to well-established preprocessing pipelines for human brain MRI data. The characteristics of brain structures across the baboon adult life course were analyzed, based on non-invasive *in vivo* MRI data. Second, we present a species-specific reference curve for brain aging in baboons, resulting from the novel adaptation of our well-established *BrainAGE* method. Using *BrainAGE*, future studies investigating experimentally induced brain changes in different programming paradigms can refer and compare to these new reference curves in normal brain volumes and individual brain aging across the adult baboon life course. Third, applying this innovative brain aging biomarker, the present study is the first to reveal modifications of individual brain aging trajectories resulting from developmental programming in a non-human primate MNR model.

The analyzes of the *in vivo* MRI data from healthy, untreated baboons showed a strong decline in GM volume and increase in WM volume during adult lifespan in males as well as females. In comparison, MRI studies in humans suggest linear decline in GM volume, non-linear age effect in WM volume decline as well as increase of CSF volume to be predominant during adulthood in both genders (Good et al., 2001; Resnick et al., 2003). In contrast, chimpanzees and rhesus monkeys show only very small (if any) age-related decline in GM and WM volumes during adulthood (Sherwood et al., 2011; Chen et al., 2013; Autrey et al., 2014). Thus, our results suggest the baboon to be the animal model closest to the human in terms of changes in brain tissue across the lifespan, and thus best suited for future translational studies.

Based on the age-related patterns of brain tissue loss, we have recently presented a fully-automatic brain age estimation framework for use in humans (Franke et al., 2010), which aggregates the complex aging patterns across the whole brain. The result is a single global estimation score of an individual “brain age” that accounts for the individual multidimensional aging pattern across the whole brain. Several studies in human samples provide evidence for the *BrainAGE* method to accurately and reliably model age-related spatiotemporal human brain changes as well as to estimate individual deviations from healthy brain aging trajectories. Moreover, *BrainAGE* results profoundly correlate with a number of general lifestyle and health parameters, disease markers, and cognitive functions (Franke et al., 2010, 2012, 2014; Gaser et al., 2013). Based on the original *BrainAGE* framework, this paper presents a species-specific adaptation for baboon brain aging, including a novel baboon-specific preprocessing tool for MRI data and validated machine learning methods for pattern recognition in order to model typical brain aging characteristics and to subsequently estimate individual brain ages. Here, this baboon-specific adaptation of the brain age estimation framework showed excellent performance in modeling baboon brain aging using *in vivo* MRI data from 15 female and 14 male baboons, aged 4–22 years.

Applying this novel, non-invasive *in vivo* MRI biomarker to a sample of baboons with 30% reduction in global maternal nutrition during pregnancy, this study shows premature brain aging of about 2.7 years in the young adult MNR female subjects (4–7 years; human equivalent 14–24 years). Several studies of fetal and postnatal MNR baboon offspring in this model have established a multi-system altered phenotype, affecting the cardiovascular system (Clarke et al., 2015), liver (Cox et al., 2006b), kidney (Cox et al., 2006a; Pereira et al., 2015), and brain (Antonow-Schlorke et al., 2011; Li et al., 2013a,b). With regards to brain development and function, a recent histological study utilizing the same baboon model of moderate MNR during pregnancy already indicated major impairments of fetal brain development, including disturbances of early organizational processes in cerebral development on a histological and gene product level, neurotrophic factor suppression, imbalances in cell proliferation and developmental cell death, impaired glial maturation and neuronal process formation, as well as altered gene expression (Antonow-Schlorke et al., 2011), resulting in an altered cognitive and behavioral phenotype during childhood and adolescence with female but not male MNR offspring demonstrating more variable and lower levels of persistence and attention and less emotional arousal than female CTR offspring by 3.3 years of age (human equivalent 11.5 years) (Keenan et al., 2013). The present neuroanatomical study on brain aging in the MNR baboon model is the first evidence in a NHP sample with MNR that appraises and quantifies the effects of fetal malnutrition on neuroanatomical aging in young adulthood, employing non-invasive *in vivo* data collection and a novel, baboon-specific evaluation approach. The increased *BrainAGE* scores in the female MNR baboons provide the first *in vivo* evidence for premature brain aging during young adulthood following impaired fetal brain development induced by moderate MNR during gestation. These results are in line with our former studies of brain development and (cognitive) function also showing stronger alterations due to MNR during gestation in the female baboon offspring (Antonow-Schlorke et al., 2011; Rodriguez et al., 2012; Keenan et al., 2013; Li et al., 2013a).

Increasing evidence from developmental programming studies often shows distinct differences between males and females (Aiken and Ozanne, 2013). Potential mechanisms for the different effects in males and females observed in this study are varying timing of puberty and adolescence in male and female baboons and its associated hormone changes. Puberty and adolescence in female baboons occur around the age of 3 to 4 years with baboons attaining adult size around 6 years of age. In contrast, puberty and adolescence in male baboons occur between 4 and 7 years, with males reaching full size around 10 years of age (Crawford et al., 1997; Jolly and Phillips-Conroy, 2003). These differing maturation trajectories probably also include brain maturation. Thus, processes of brain tissue gain as a result of pre-pubertal growth and pruning may still be continuing in the male subjects studied, whereas in the female subjects brain maturation was closer to completion, such that the processes of age-related tissue loss occurring during adulthood had already started. For future studies we propose to continue to apply this *in vivo* biomarker for brain aging in the same sample of MNR offspring during middle and late adulthood in order to comprehensively track the long-lasting effects of developmental programming on gender-specific alterations of individual brain aging trajectories.

Several established models for brain aging in humans showed significant relationships between individual brain aging and health and lifestyle variables as well as medical drug use (Franke et al., 2014; Habes et al., 2016). Delayed brain aging has been found to be associated with higher levels of education and physical activity (Steffener et al., 2016) as well as higher levels of meditation practice (Luders et al., 2016). Furthermore, advanced brain aging was shown to be indicative of poorer physical fitness, lower fluid intelligence, higher allostatic load, and increased mortality (Cole et al., 2017), and even predicting the onset of cognitive decline (Franke et al., 2012; Gaser et al., 2013). Additionally, a recent study on changes of individual *BrainAGE* during the course of the menstrual cycle in humans (Franke et al., 2015) proves the *BrainAGE* method being capable of reliably indicating even temporary neuroanatomical changes as for example occurring during the course of the menstrual cycle. Consequently, the *BrainAGE* method offers new approaches to monitor subtle neuroanatomical changes in longitudinal intervention and treatment studies in humans and experimental animal models, e.g., exploring the effects of daily activity, protective nutrients, or medication on individual brain structure.

In conclusion, to our knowledge this is the first study to utilize the power of MRI brain imaging in a NHP model of controlled decreased fetal nutrition and MNR. We have developed a novel, species-specific, non-invasive *in vivo* MRI biomarker for brain aging that shows great potential for further studies in our well-established NHP model of developmental programming and aging. For example, these offspring will be maintained to follow the aging process over the rest of their life course. The brain aging biomarker enables a well-controlled, repeatable, and non-invasive *in vivo* exploration of individual effects on subtle, yet clinically-significant, changes in brain structure affecting neuroanatomical maturation and aging due to various environmental challenges experienced in human pregnancy (e.g., maternal obesity and diabetes, effects of maternal stress and placental insufficiency). Future studies can combine this MRI methodology with cognitive and behavioral studies, as well as treatment and intervention studies. Additionally, there is a clear need for gender-specific mechanisms, such as those shown here, to be taken into account in future studies. We observed premature brain aging in young adult female offspring. Furthermore, a species-specific and well-performing adaptation of the *BrainAGE* method for analyzing brain aging in rodents has recently been presented (Franke et al., 2016), thus also enabling future (longitudinal) studies of experimentally induced changes in brain maturation and aging in rodent models. In summary, the *BrainAGE* method can potentially identify a variety of environmental factors and mechanisms that induce premature brain atrophy at an individual level and contribute to a better understanding of healthy and pathological brain aging in animal models and humans.

## Author contributions

KF planned study design, developed part of the methods, performed statistical analyses, interpreted results, wrote manuscript. GC planned experimental design, acquired MRI data. RD developed part of the methods. CG contributed to methods development and manuscript. AK took care of animals, planned experimental design, acquired MRI data. CL took care of animals, planned experimental design, acquired MRI data. MS contributed to study design and manuscript. PN contributed to study design, experimental design, and manuscript.

## Funding

This work was supported in part by the European Community [FP7 HEALTH, Project 279281 (BrainAge) to KF], the German Research Foundation [DFG, Project FR 3709/1-1 to KF], and the National Institute of Health [K25 DK089012 & R24 RR021367 to PN]. The sponsors had no role in the design and conduct of the study; collection, management, analysis, and interpretation of the data; and preparation, review, or approval of the manuscript.

### Conflict of interest statement

The authors declare that the research was conducted in the absence of any commercial or financial relationships that could be construed as a potential conflict of interest.
